# Isolation and Molecular Evidence of Tunisian Sheep-like Pestivirus (*Pestivirus N*) in Persistently Infected Sheep in Northern Italy, 2023

**DOI:** 10.3390/v16060815

**Published:** 2024-05-21

**Authors:** Enrica Sozzi, Gabriele Leo, Fatbardha Lamcja, Massimiliano Lazzaro, Cristian Salogni, Davide Lelli, Cristina Bertasio, Giulia Magagna, Ana Moreno, Giovanni Loris Alborali, Moira Bazzucchi, Antonio Lavazza

**Affiliations:** 1 Istituto Zooprofilattico Sperimentale della Lombardia e dell’Emilia Romagna “Bruno Ubertini” (IZSLER), Via Antonio Bianchi 7/9, 25124 Brescia, Italy; gabriele.leo@izsler.it (G.L.); cristian.salogni@izsler.it (C.S.); davide.lelli@izsler.it (D.L.); cristina.bertasio@izsler.it (C.B.); giulia.magagna@izsler.it (G.M.); anamaria.morenomartin@izsler.it (A.M.); giovanni.alborali@izsler.it (G.L.A.); moira.bazzucchi@izsler.it (M.B.); antonio.lavazza@izsler.it (A.L.); 2 Dipartimento Veterinario e Sicurezza degli Alimenti di Origine Animale ATS, Brescia, Viale Duca degli Abruzzi 15, 25124 Brescia, Italy; fatbardha.lamcja@ats-brescia.it (F.L.); massimiliano.lazzaro@ats-brescia.it (M.L.)

**Keywords:** Tunisian sheep-like pestivirus, sheep, phylogenetic analysis, Italy

## Abstract

Over the last few decades, several pestiviruses have been discovered in ruminants, pigs, and, more recently, in non-ungulate hosts. Consequently, the nomenclature and taxonomy of pestiviruses have been updated. The Tunisian sheep-like pestivirus (TSV, *Pestivirus N*) is an additional ovine pestivirus genetically closely related to classical swine fever virus (CSFV). In this study, during a survey of pestivirus infections in ovine farms in the Lombardy region of Northern Italy, we identified and isolated a pestivirus strain from a sheep that was found to belong to *Pestivirus N* species based on its genomic nucleotide identity. The sheep itself and its lamb were found to be persistently infected. We performed molecular characterization and phylogenetic analysis of three viral genomic regions (a fragment of 5′-UTR, partial N^pro^, and the whole E2 region). In conclusion, these results confirmed circulating TSV in Northern Italy after notification in Sicily, Italy, and France. Correlation with Italian, Tunisian, and French strains showed that detection might have resulted from the trading of live animals between countries, which supports the need for health control measures.

## 1. Introduction

Pestivirus is a genus of enveloped viruses belonging to the family Flaviviridae. Pestiviruses comprise pathogens of livestock, such as classical swine fever virus (CSFV—*Pestivirus suis*) and bovine viral diarrhea virus (BVDV-1—*Pestivirus bovis* and BVDV-2—*Pestivirus tauri*), that cause diseases of outstanding economic relevance notifiable to the World Organisation for Animal Health (WOAH) [1,2]. Owing to the increasing number of diverse pestiviruses identified and characterized, the taxonomy of the genus Pestivirus was revised in 2017 [3] based on the nucleotide or amino acid sequence distances of complete coding sequences, in combination with antigenic differences, natural host range, and pathology. Consequently, pestiviruses are classified into 11 species, designated *Pestivirus A* through *Pestivirus K*. Since then, multiple new pestiviruses have been reported, including pathogens associated with diseases in pigs and small ruminants. Therefore, Postel et al. [4] proposed expanding the number of pestivirus species to 19 by adding 8 additional species designated *Pestivirus L* through *Pestivirus S*. Recently, the International Committee on Taxonomy of Viruses (ICTV) *Flaviviridae* Study Group proposed to change the names for the species within the family Flaviviridae following the ICTV decision to change all established species names to a new standardized binomial format [5]. Therefore, the eleven current recognized species were renamed to comply with the binomial format, as shown in Table 1.

The new taxonomy release, approved by the ICTV in July 2022 and rectified in March 2023 (MSL #38), includes the 19 species mentioned above in the *Pestivirus* genus. However, only the first eleven established species were named using the binomial format, whereas the last nine are still named *Pestivirus* from *L* to *S*.

The Tunisian sheep-like pestivirus (TSV, *Pestivirus N*) was first isolated from batches of a contaminated sheep pox vaccine in Tunisia and represents an additional ovine pestivirus that is genetically and antigenically more closely related to CSFV than to the border disease virus (BDV, *Pestivirus ovis*) [6,7,8]. Subsequently, TSV was detected in sheep in France and goats and sheep in Italy [9,10]. Other pestiviruses from sheep and goats are closely related to CSFV, such as the Aydin-like pestivirus (*Pestivirus aydinense*) and ovine/IT pestivirus ovIT PeV (*Pestivirus O*), which can significantly interfere with the serological diagnosis of CSFV [11]. A phylodynamic study showed that CSFV, affecting pigs (*Sus scrofa*), and ovIT PeV, affecting sheep (*Ovis aries*), which possibly shared TSV as a common ancestor > 200 years ago [12]. In small ruminants, the natural hosts, TSV, can cause mild fever and leukopenia three to six days post-infection [13]. Infection of pregnant ewes with TSV resulted in clinical signs typically associated with border disease, including high rates of abortion, stillbirth, and birth of weak and hairy-shaker lambs [6]. Recently, considering the close relationship between TSV and CSFV, pigs were found to be susceptible to TSV; however, they did not show any clinical sign after horizontal transmission [8].

The pestivirus genome consists of a single-stranded positive-sense RNA approximately 12.3 kb in length. A single open reading frame encodes four structural proteins (C, Erns, E1, and E2) and seven non-structural proteins (N^pro^, p7, NS2-3, NS4A, NS4B, NS5A, and NS5B) and is flanked by 5′- and 3′-untranslated regions (UTRs). Phylogenetic studies generally classify virus isolates based on sequences generated from 5′-UTR and N^pro^ genomic regions. Based on this genetic classification and recent publications, we report the detection of a pestivirus strain identified from samples collected from a sheep and its lamb held on pasture in northern Italy. We sequenced the E2 region and performed corresponding phylogenetic analyses to better describe such a viral strain. Based on its genomic nucleotide identity, it was found to belong to the *Pestivirus N* species.

## 2. Materials and Methods

### 2.1. Samples

In April 2023, within the survey to determine the prevalence of pestivirus infections in ovine farms in the Lombardy region of Northern Italy, we analyzed 87 sera for detecting both pestivirus antigens and antibodies. Samples were collected from the same wandering flock of around 2000 animals. For this project on pestivirus epidemiology, authorized and founded by the Italian Ministry of Health, we take advantage of testing those available sera that have been taken within the framework of the national plan for eradicating brucellosis in sheep and goat herds. One young female sheep (ID77) gained our attention as it tested positive in real-time RT-PCR for pestivirus detection [14] but gave a negative result in the homemade pestivirus antibody competitive ELISA test.

To verify whether the sheep was immunotolerant to the virus and considered a persistently infected animal, subsequent samples were obtained as follows:

- In November 2023, two blood samples were collected after the mountain pasture, respectively, with (i.e., ethylenediaminetetraacetic acid—EDTA anticoagulated) and without anticoagulated factors.
- In January 2024, the ID77 sheep delivered at full term to a single, live, viable female lamb, which showed no clinical signs of any small ruminant disease. One week after delivery, a third sampling was performed, including two blood samples with and without anticoagulant, milk, nasal and rectal swabs of the sheep, and nasal and rectal swabs of the lamb (ID01).

### 2.2. Serological Analysis

Pestivirus antibody was detected by a homemade competitive enzyme-linked immunosorbent assay (ELISA) using horseradish peroxidase (HRP)-conjugated monoclonal antibodies and the recombinant NS3 protein as an antigen [15]. This test is regularly used in IZSLER’s diagnostic routine. The MAb 3H4 is adsorbed onto a Nunc^®^MaxiSorp—microplate (Thermo Fisher Scientific Inc., Waltham, MA, USA) at a saturating concentration to trap the recombinant NS3 antigen. Sera diluted 1/50 are dispensed in duplicate wells, one containing antigen and the other not (only buffer). The specificity of the recombinant NS3 protein-based ELISA was confirmed using specific anti-CSFV and anti-BVDV sera of immunized swine and bovine, respectively. After incubation and washing, a pre-determined optimal dilution of an MAb (1G10) anti-ruminant immunoglobulin G (IgG), peroxidase-conjugated, was added. After the development of the reaction, results were reported as S/P values, calculated as the ratio between the net optical density (OD) of the sample (obtained by subtracting the OD of the control well without antigen from the OD of the well with antigen) and the net OD of a positive control serum tested in each plate, with the positive cut-off value for serum samples set at 0.1. In addition, E2-specific antibodies were analyzed by ELISA using the commercial CSFV Ab test (IDEXX Laboratories, Liebfeld, Switzerland) according to the manufacturer’s recommendations. The samples were considered positive when the blocking percentage was ≥40%.

We decided to use the commercial test as a proxy to detect CSFV-cross-reacting antibodies following the indication that (1) CSFV originated about 225 years ago from a Tunisian sheep-like pestivirus, thus conserving a certain cross-reactivity [16]; (2) the CSFV antibody-specific ELISA can detect antibodies induces by another pestivirus, the ovine pestivirus ovIT PeV (*Pestivirus O*) [17], which itself emerged from TSV; (3) the same commercial test has been used to detect CSFV-cross reacting antibodies produced after experimental infection of sheep and pigs with ovIT PeV [12,17].

### 2.3. Virological Analysis

Virological analyses were performed on these samples: (i) serum obtained in April 2023, (ii) serum and EDTA-blood samples collected in November 2023, (iii) serum, EDTA-blood, nasal and rectal swabs collected in January 2024 of the sheep ID77, and (iv) the nasal and rectal swabs obtained from the lamb ID01 in January 2024. Nasal and rectal swabs were homogenized in 1 mL of in minimum essential medium (MEM; Gibco, Life Technologies, Paisley, UK) supplemented with an antibiotic (1000 U/mL penicillin, 1 mg/mL streptomycin; Gibco, Life Technologies, Paisley, UK) and anti-mycotic (2.5 μg/mL amphotericin B; Gibco, Life Technologies, Paisley, UK). After centrifugation, the supernatant was analyzed. The samples mentioned above were inoculated onto monolayers of primary bovine fetal kidney, Madin–Darby bovine kidney (MDBK), and swine testicular (ST) cell lines for viral isolation. Additionally, primary lamb kidney (LKi) cells were inoculated only with specimens from the third sampling. After two hours at 37 °C in a humidified 5% CO_2_ incubator, the inoculum was removed, and minimum essential medium (MEM; Gibco, Life Technologies, Paisley, UK) supplemented with 1% gamma-irradiated (25.0–40.0 kGy) fetal bovine serum, free of pestivirus antibodies, antigens, and genome, and 1% penicillin-streptomycin (1000 U/mL penicillin, 1 mg/mL streptomycin; Gibco, Life Technologies) was added. The inoculated cell monolayers were observed daily for 5–7 days for the appearance of a cytopathic effect (CPE) and then further subcultured to improve the chance of isolation until the fourth passage, after which cell lysates were assessed by molecular methods (see Section 2.4). In addition, according to the routine procedure applied in our lab, each passage of the inoculated cell culture monolayer was tested using the IDEXX BVDV Ag/Serum Plus kit (HerdChek, IDEXX, Hoofddorp, The Netherlands) to assess the incremental OD and thus confirming the isolation.

### 2.4. Viral RNA Extraction and Real-Time RT-PCR for Pestiviruses Screening

After cell lysates centrifugation at 3000× *g* for 15 min, RNA was extracted using a Biosprint 96 One-For-All Vet Kit (Qiagen, Hilden, Germany) on a Thermo Fisher Scientific KingFisher Apex Purification System (Waltham, MA, USA) according to the manufacturer’s instructions. Pestiviruses were detected using a real-time RT-PCR screening method, following the procedure previously described [14]. This method is routinely used in many diagnostic laboratories to detect the most relevant species of the genus Pestivirus (*Pestivirus bovis*, *Pestivirus tauri*, *Pestivirus suis*, *Pestivirus ovis*). Being based on the amplification of the 5′ non-translated region (5′ NTR), it is considered a pan-pestivirus assay. Positive control was represented by *Pestivirus bovis*, BVDV NADL-strain cryolysate suitably diluted to give a Ct value of 30 ± 3.

### 2.5. PCR Amplification and Sequencing

The sample obtained in April 2023 that tested positive to the real-time RT-PCR pestiviruses screening (124910/77-2023) was subsequently characterized by Sanger sequencing using RT-PCR to amplify a fragment from the 5′-UTR region [18] and from the N^pro^ region [19]. Additionally, the complete E2 gene [20] was amplified for sequencing. Briefly, the reactions were performed using a One-Step RT-PCR Kit (Qiagen). The PCR reaction was carried out in 25 µL total volume, containing 1.2 µL One-Step RT-PCR Enzyme Mix, 5 µL 5× Buffer, 2.5 µL template RNA, 0.25 µL RNase inhibitor (40 U/µL), 1 µL 10 mM dNTPs, the following PCR primers a final concentration of 0.7 µM of 5′–TGGTGGCCTTATGAGAC-3′ (E2F) [20] and 5′-CCCATCATCACTATTTCMCC-3′ (P7R, modified from Lang et al. [20], and 11.55 µL RNase-free water. RT-PCR was performed with the following thermal cycle: 45 °C for 30 min to synthesize the first strand cDNA, 95 °C for 15 min to activate the HotStarTaq DNA Polymerase, and 45 cycles of 15 s at 94 °C, 30 s at 54 °C, and 90 s at 72 °C. These cycles were followed by a final extension period of 10 min at 72 °C. The 5′-UTR and N^pro^ PCR products were purified using the QIAquick PCR Purification Kit (Qiagen, Hilden, Germany). Sequencing reactions were performed using the BigDye Terminator v.1.1 kit (Thermo Fisher Scientific, Waltham, MA, USA) and analyzed using an ABI Prism 3730 DNA Analyser (Applied Biosystems). For E2 sequencing following gel electrophoresis, the PCR products were purified by gel extraction with NucleoSpin Gel and PCR Clean-up (Macherey-Nagel, Düren, Germany) according to the manufacturer’s instructions. Cycle sequencing was performed using 4 µL DNase-RNase-free water, 2 µL BigDye™ Terminator v3.1 Cycle Sequencing (Thermo Fisher Scientific, Waltham, MA, USA), 2 µL 5× Sequencing Buffer (Thermo Fisher Scientific, Waltham, MA, USA), 1 µL primer (1.6 µM), and 1 µL PCR product. Subsequently, the products were purified using the BigDye Xterminator™Purification Kit (Thermo Fisher Scientific, Waltham, MA, USA) according to the manufacturer’s instructions, and the gene was sequenced on an Applied Biosystems Seqstudio Genetic Analyser (Thermo Fisher Scientific, Waltham, MA, USA) using the long_BDX run module. Additionally, the 5′-UTR region obtained in January 2024 from the serum of sheep ID77 and the nasal swab of lamb ID01 was sequenced to further confirm the persistence of the viral strain within the same flock [18].

### 2.6. Phylogenetic Analysis

The sequences were analyzed using DNAStar v.17. Nucleotide sequences were aligned using ClustalW with respect to 47, 37, 21 (5′-UTR, N^pro^, and E2) representative pestivirus strains available in GenBank. Manual editing was performed using MEGA v.X [21]. Phylogenetic analysis was performed by comparing the aligned sequences, as described above. Three phylogenetic trees were constructed using the Maximum Likelihood method in MEGA v.X software. Bootstrap analysis was performed using 1000 replicates using the Kimura 2 parameter (K2), Tamura-Nei (TN93) and General Time Reversible models (only G for 5′-UTR, G + I for N^pro^, and E2), respectively for 5′-UTR, N^pro^, and E2 that were identified using ModelFinder selection.

Nucleotide identity percentages were determined by NCBI BLASTn analysis.

The estimation of Evolutionary Divergence between Sequences (pairwise distances) was conducted using the Kimura 2-parameter, Tamura-Nei, and General Time Reversible models for 5′-UTR, N^pro^, and E2, respectively, using Bootstrap 1000. All analyses were performed in MEGA v.X.

### 2.7. Nucleotide Sequence Accession Numbers

The three viral genomic sequences (a fragment of the 5′-UTR, the partial N^pro^, and the whole E2 region) of the first serum sample 124910/77-2023 were deposited in GenBank (NCBI) with the accession numbers PP236840, PP236841, and PP236842 respectively.

## 3. Results

The 124910/77-2023 serum sample tested positive with the real-time RT-PCR pestiviruses screening, and a viral strain was identified. The specific products of the 5′-UTR region fragment (218 bp), the partial sequence of the N^pro^ region (405 bp), and the complete E2 region (1212 bp) were obtained. All following samples—EDTA anticoagulated blood and serum samples (November 2023) and EDTA anticoagulated blood, serum, milk, nasal, and rectal swabs of sheep and nasal and rectal swabs of lamb (January 2024) were positive in pestivirus screening with different Ct values (Table 2). In contrast, serological investigations always yielded negative results using both the homemade pestivirus recombinant NS3 protein ELISA and the commercial kit for CSFV E2-specific antibodies.

**Figure 1:**
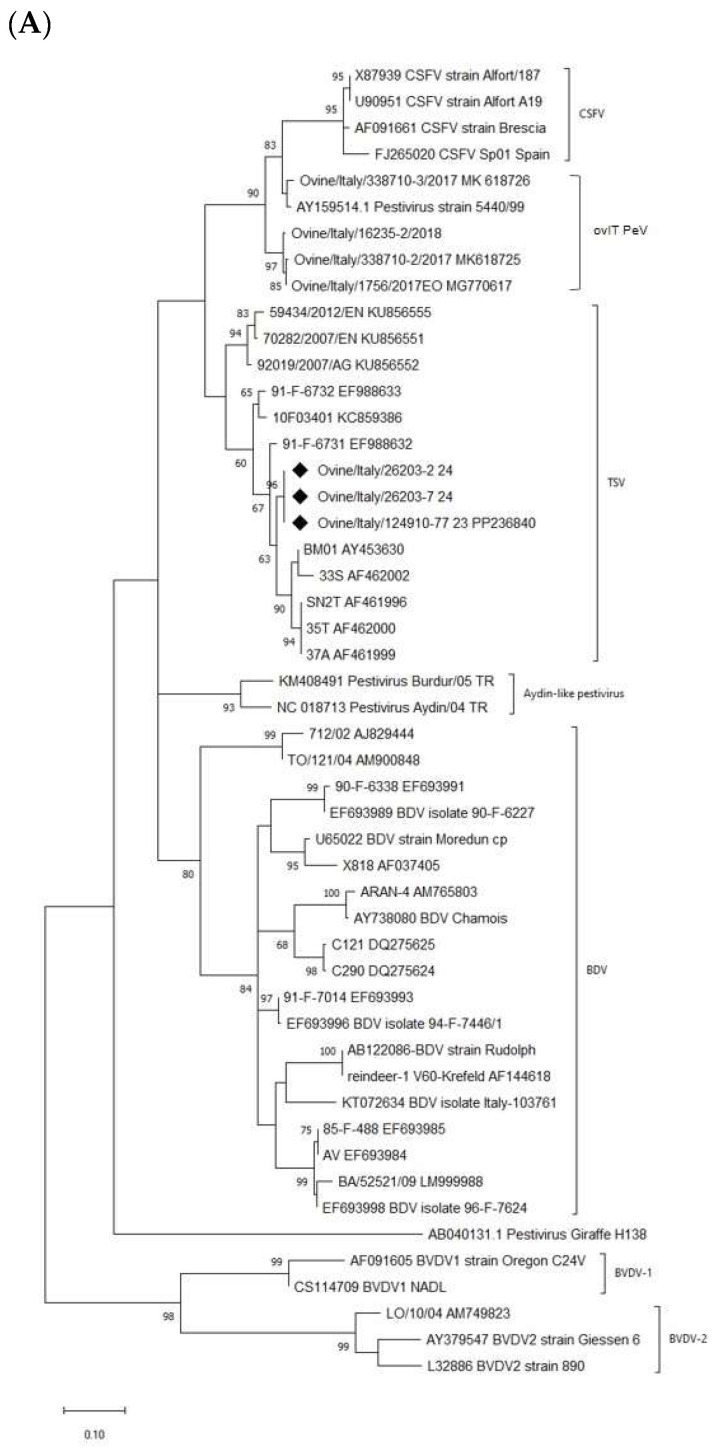

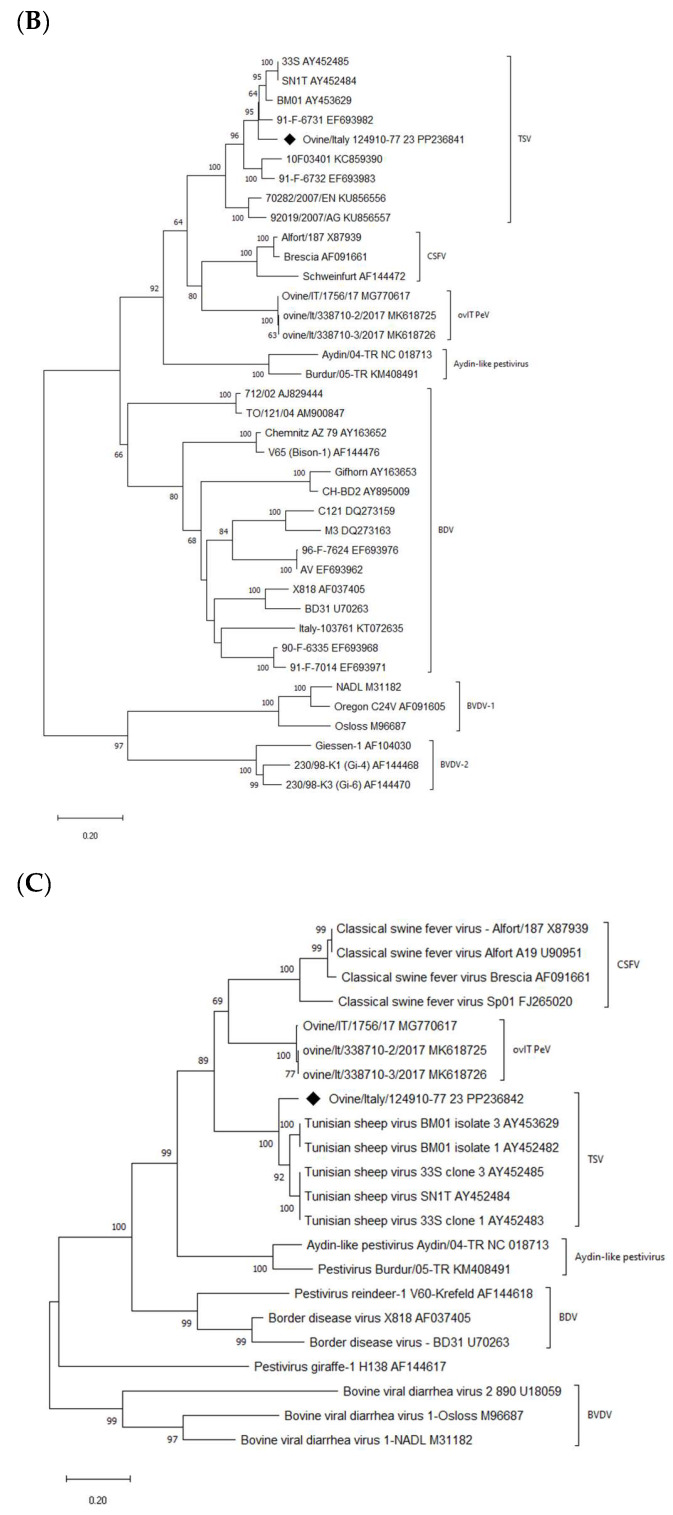
The phylogenetic trees are based on (**A**) the partial 5′-UTR, (**B**) partial N^pro^, and (**C**) complete E2 regions sequencing of the TSV strain detected in the survey and the TSV isolates and other pestivirus sequences deposited in GenBank. Molecular analyses were performed with MEGA v.X software with bootstrap analysis (1000 replicates) using the Maximum Likelihood method based on the Kimura 2 parameter (G), Tamura-Nei (G + I), and General Time Reversible (G + I) models, respectively, for 5′-UTR, N^pro^, and E2. Bootstrap values > 60% are shown. Sequences generated in this study are indicated with a black rhombus. Published sequences are identified by strain and GenBank accession number.

An alignment for each analyzed region was built, including sequences representative of certain pestivirus species retrieved from the GenBank database. Alignment files were used for both the phylogenetic analysis and the percentage similarity of the pairwise distance calculation. Phylogenetic trees revealed different branches corresponding to pestivirus species BVDV-1, BVDV-2, BDV, CSFV, ovIT PeV, Aydin-like pestivirus, and Giraffe strains. In the present study, the ovine pestivirus (124910/77-2023) was clustered in the Tunisian sheep-like pestivirus group (Figure 1). Bootstrap analysis strongly supported this result, thus indicating its identity as a *Pestivirus N* species. No correlation was observed between the pestivirus isolated from sheep in this study and the pestivirus strains previously isolated from small ruminants in Italy, such as BDV and ovIT PeV. The analyses of the other considered genomic regions confirmed this result. Italian 124910/77-2023 strain showed the highest nucleotide identities with Tunisian strains (90.6–97.2%, 82.9–92.8%, and 87.9–88.1% for the 5′-UTR, N^pro^, and E2 regions, respectively) with the highest value with the French isolate 91-F-6731 (97.2% and 92.8% for the 5′-UTR and N^pro^ region, respectively).

The Tunisian isolates were divided into two clusters: the first included Italian field isolates identified in Sicily. The second included (i) strains isolated from vaccine preparations (the *Pestivirus N* was first isolated from different batches of a contaminated Tunisian sheep pox vaccine), (ii) French strains, and (iii) the new Italian strain identified in this study. Furthermore, the subdivision of the Tunisian isolates into two separate branches, as determined by the analysis of the 5′-UTR, was also supported by phylogenetic analysis of the N^pro^ coding region. Other 5′-UTR short fragments were obtained in sheep serum and lamb rectal swab samples (26203/2-2024 and 26203/7-2024). These sequences showed 100% identity to the 124910/77-2023 strain.

For viral isolation at 5–7 days post-infection (dpi), culture supernatants were frozen at −20 °C, and then the cryo-lysates passage thrice. Since no CPE was observed in any passages, the cryolysate from the last passage was screened by the pestiviruses’ real-time RT-PCR. *Pestivirus N* was successfully isolated from the serum sample taken in November 2023 in the MDBK cell line, while no viral replication was detected in the ST and primary bovine fetal kidney cell lines. To confirm viral isolation, samples were passaged four times, and each passage was tested in an Ag ELISA using the IDEXX BVDV Ag/Serum Plus kit (HerdChek, IDEXX, Hoofddorp, The Netherlands). Increasing Optical Density (OD) was observed. In addition, the sequencing analysis of the partial 5′-UTR region conducted on the fourth passage of the MDBK cell lysate confirmed the viral isolation.

Virological results for samples obtained from the sheep and her lamb in January 2024 were the following: *Pestivirus N* strains were isolated on LKi and ST cells from EDTA-blood, serum, and nasal swabs of sheep ID77 and rectal swabs of lamb ID01. Conversely, the rectal swab of the sheep and the nasal swab of the lamb resulted in a negative result on any cell culture employed for viral isolation. No viral replication was detected in the primary bovine fetal kidney and MDBK cell lines for any of these samples (Table 2).

## 4. Discussion

Pestiviruses are enveloped viruses with highly variable, single-stranded, positive-sense RNA genomes. Therefore, several new genetically distinct groups of pestiviruses have emerged in domestic livestock and wild animals, leading to the proposal of additional pestivirus species. A pestivirus strain was isolated from live sheep in 2023 during pestivirus surveillance of small ruminants in Lombardy in Northern Italy. Based on its genomic nucleotide identity, it belongs to the *Pestivirus N* species. We could not identify the origin of the infection; since the affected sheep was born on the farm in May 2022, it could be only hypothesized that the viral transmission occurred directly through the transport of live infected animals or indirectly due to contaminated equipment’s as has been previously shown for other pestiviruses, such as BVDV, CSFV, or Bungowannah virus (BungoV) [22,23,24]. In this study, TSV excretion in nasal secretions, milk, and rectal swabs was observed, supporting the potential role of these materials in direct or indirect horizontal transmission.

To date, fragments of the 5′-UTR (GenBank PP236840), partial N^pro^ encoding region (GenBank PP236841), and complete E2 region (GenBank PP236842) were used to determine the genetic relatedness between 124910/77-2023 strain and other pestiviruses. The sequencing of specific TSV regions obtained in this study revealed a significant % nucleotide identity with published TSV strains ranging between 90.6% and 97.2% in the 5′-UTR region. The analysis of both the partial N^pro^ and the complete E2 regions confirmed the results obtained with 5′-UTR region analysis, clustering the new Italian sheep strain within the Tunisian strains cluster. However, none of the previously identified TSV strains was identical enough to establish a real origin of the strain identified here, and it was clearly separated from Sicilian strains and more similar to the French ones.

The sheep tested negative for antibodies against pestiviruses over the long term, and the serological analysis also confirmed the lack of antibodies against CSFV. Therefore, it was clear that the tested sheep, ID77, was an immunotolerant, persistently viraemic animal. Subsequently, additional sampling was performed first to confirm persistent viremia and then, after delivery, to verify viral excretion from the sheep (milk, nasal, and rectal swabs as well as blood) and lamb (nasal and rectal swabs as well as blood). Indeed, TSV was identified in all these samples and successfully isolated from the sheep’s nasal swab and the lamb’s rectal swab.

This study confirmed that TSV replicates efficiently in LKi cells, as previously demonstrated by the fact that this virus is well adapted to ovine cells [8]. Although less efficient, TSV was also grown on porcine ST and bovine MDBK cells (only for serum samples obtained in November 2023). Moreover, no viral replication was detected in primary bovine fetal kidney cells. Although the serum collected in November 2023 and the nasal swab sampled in January 2024 of the sheep showed high Ct values (35.33 and 27.34, respectively), the *Pestivirus N* from these samples was efficiently cultivated on cell culture. Instead, *Pestivirus N* from other samples, i.e., the serum of sheep collected in April 2023 and the nasal swab of the lamb sampled in January 2024 with lower Ct values (22.29 and 25.58, respectively), was not reproduced in culture. This was unsurprising since the amount of viral RNA measured in a sample does not necessarily correlate directly to the viral infectivity. Some factors, such as sampling, storage, transport, and processing, may hurt the vitality/infectivity of virions. This may explain why the *Pestivirus N* from the nasal swab of the lamb was not isolated on the different cell cultures used. In addition, in April 2023, the laboratory did not have the LKi and ST cell lines available, which are more sensitive for the isolation of *Pestivirus N* [10]. When these were provided, the sample was unfortunately exhausted. Interestingly, the *Pestivirus N* was efficiently isolated on the MDBK cells from the serum sample of the sheep collected in November 2023 but not from the later samples, which showed low Ct values. This may be due to the sensitivity of the cell line in relation to the number of passages and laboratory processing.

To characterize the Tunisian pestivirus strains obtained in 2024, we preliminarily performed the amplification and sequencing of the 5′-UTR from sheep serum and lamb rectal swab samples (26203/2-2024 and 26203/7-2024). The nucleotide sequences obtained were aligned with the corresponding published sequences of pestivirus reference strains. Phylogenetic analysis showed that these strains had 100% nucleotide identity with the 124910/77-2023 strain. The three TSV isolates, Aydin-like pestivirus strains, and novel ovIT PeVs formed three distinct groups located between CSFV and BDV [8]. In addition, the phylogenetic analysis showed that TSV forms two distinct groups and is more closely related to CSFV than BDV or other ruminant pestiviruses [6,7,8].

Italy has a high prevalence of small ruminant herds, and since it can host different pestiviruses, characterizing novel pestiviruses, either genetically or antigenically, and evaluating their host range is highly important [25,26,27]. Concurrently, detecting persistently infected animals that have a major impact on the transmission and spread of infection is essential. These animals are viraemic, do not have specific antibodies, and can constantly excrete remarkable amounts of viruses even without clear signs of disease. In the case under investigation, the veterinarian and owner reported that no outbreaks or cases of the disease had been recorded on the farm. Surveillance of the farm to assess the serostatus of the entire sheep herd and the possible presence of TSV in other animals is underway.

Only a few complete TSV genome sequences are available; therefore, phylogenetic studies on TSV are limited to analyzing partial genomic and deduced amino acid sequences. The next step in our research will be to carry out a complete genome characterization of the Italian TSV strain.

Although TSV is related to CSFV, Meyer et al., 2021 [8] showed that experimental infection in pigs did not result in clinical signs. However, the TSV genome was detected in the blood at 5, 7, and 14 dpi, confirming that TSV could replicate in domestic pigs under experimental conditions. Therefore, this ruminant pestivirus is a candidate for a switch to porcine hosts after ongoing viral evolution, which would have severe consequences for the serological diagnosis of classical swine fever, potentially affecting the control and monitoring programs performed in many parts of the world.

## 5. Conclusions

Several new, genetically diverse groups of pestiviruses have emerged in domestic and wild animals, adding to the continuously growing list of approved and tentative pestivirus species.

Phylogenetic analyses of short partial genome sequences identified a Tunisian sheep-like pestivirus recently circulating in sheep in northern Italy. This is the first case in the north of Italy, and further investigation is required to understand how it presents and spreads in Italian sheep herds.

Similarly, further epidemiological studies are needed to understand the role of different wild and domestic species in spreading and maintaining TSV infections.

## Figures and Tables

**Table 1:** Previous and new taxonomy of established pestivirus species to comply with the ICTV-mandated binomial format.

PREVIOUS TAXONOMY				NEW TAXONOMY
Family	Genus	Species	Abbreviation	Species
*Flaviviridae*	*Pestivirus*	*Pestivirus A*	BVDV-1	*Pestivirus bovis*
*Flaviviridae*	*Pestivirus*	*Pestivirus B*	BVDV-2	*Pestivirus tauri*
*Flaviviridae*	*Pestivirus*	*Pestivirus C*	CSFV	*Pestivirus suis*
*Flaviviridae*	*Pestivirus*	*Pestivirus D*	BDV	*Pestivirus ovis*
*Flaviviridae*	*Pestivirus*	*Pestivirus E*	PHV	*Pestivirus antilocaprae*
*Flaviviridae*	*Pestivirus*	*Pestivirus F*	BuPV	*Pestivirus australiaense*
*Flaviviridae*	*Pestivirus*	*Pestivirus G*	-	*Pestivirus giraffae*
*Flaviviridae*	*Pestivirus*	*Pestivirus H*	HoBi	*Pestivirus brazilense*
*Flaviviridae*	*Pestivirus*	*Pestivirus I*	-	*Pestivirus aydinense*
*Flaviviridae*	*Pestivirus*	*Pestivirus J*	NrPV	*Pestivirus ratti*
*Flaviviridae*	*Pestivirus*	*Pestivirus K*	APPV	*Pestivirus scrofae*

**Table 2:** PCR testing results and virus isolation from the sheep and lamb samples examined in this study (n.d.—not done).

					Isolation on Cell Culture			
Strain	Host	Specimen	Date of Sampling	Real Time RT-PCR (Ct Value)	MDBK	Primary Bovine Foetal Kidney	Lki	ST
124910/77	sheep ID77	serum	April 2023	22.29	no	no	n.d	n.d
373570-1	sheep ID77	serum	November 2023	35.33	yes	no	n.d	no
373570-2	sheep ID77	EDTA-blood	November 2023	35.35	n.d	n.d	n.d	n.d
26203-1	sheep ID77	EDTA-blood	January 2024	21.95	no	no	yes	yes
26203-2	sheep ID77	serum	January 2024	20.26	no	no	yes	yes
26203-3	sheep ID77	milk	January 2024	24.88	n.d	n.d	n.d	n.d
26203-4	sheep ID77	nasal swab	January 2024	27.34	no	no	yes	yes
26203-5	sheep ID77	rectal swab	January 2024	32.99	no	no	no	no
26203-6	lamb ID01	nasal swab	January 2024	25.58	no	no	no	no
26203-7	lamb ID01	rectal swab	January 2024	24.19	no	no	yes	yes

## Data Availability

The data presented in this study are available on request. The sequences obtained in this study are available in GenBank under accession numbers.
